# Health Care Support Issues for Internationally Adopted Children: A Qualitative Approach to the Needs and Expectations of Families

**DOI:** 10.1371/journal.pone.0031313

**Published:** 2012-02-20

**Authors:** Olivier Lesens, Anna Schmidt, Florence De Rancourt, Véronique Poirier, André Labbe, Henri Laurichesse, Laurent Marty, Jean Beytout, Philippe Vorilhon

**Affiliations:** 1 Clermont Université Laboratoire Microorganismes: Génome Environnement (LMGE), Université d'Auvergne, Clermont-Ferrand, France; 2 Service des Maladies Infectieuses et Tropicales, Hôpital Gabriel Montpied, Clermont-Ferrand, France; 3 Department of General Medicine, Univ Clermont, Faculté de Médecine de Clermont Ferrand, Clermont-Ferrand, France; 4 Unité de réanimation néonatale et pédiatrique, Clermont-Ferrand, France; UCL Institute of Child Health, University College London, United Kingdom

## Abstract

**Background:**

Families of internationally adopted children may face specific problems with which general practitioners (GPs) may not be familiar. The aim of the study was to explore problems faced by families before, during and after the arrival of their internationally adopted child and to assess the usefulness of a specific medical structure for internationally adopted children, which could be a resource for the GP.

**Methodology/Principal Findings:**

We conducted a qualitative study using individual semistructured guided conversations and interviewed 21 families that had adopted a total of 26 children internationally in the Puy de Dome department, France, in 2003. Quantitative data were used to describe the pathologies diagnosed and the investigations performed.Our study showed that the history of these families, from the start of the adoption project to its achievement, is complex and warrants careful analysis. Health-care providers should not only consider the medical aspects of adoption, but should also be interested in the histories of these families, which may play a role in the forming of attachments between the adoptee and their adoptive parents and prevent further trouble during the development of the child. We also showed that adoptive parents have similar fears or transient difficulties that may be resolved quickly by listening and reassurance. Most such families would support the existence of a specific medical structure for internationally adopted children, which could be a resource for the general practitioner. However, the health-care providers interviewed were divided on the subject and expressed their fear that a special consultation could be stigmatizing to children and families.

**Conclusions/Significance:**

A specific consultation with well-trained and experienced practitioners acting in close collaboration with GPs and paediatricians may be of help in better understanding and supporting adopted children and their families.

## Introduction

Currently, one child out of every 100 in France is adopted, and 80% of adoptions are international [1]. In 2010, 3504 children were adopted internationally in France, and they originated from 67 different countries, mainly Haiti (28%), Vietnam (13%), Colombia (11%), Ethiopia (10%) and China (3%) [1]. In the USA, the 1.5 million adopted children comprise 2% of American children [2]. Many medical problems that the children present on arrival are benign (dermatosis, diarrhoea) but some may spread to the rest of the family (e.g. hepatitis A) or become chronic because of misdiagnosis (e.g., hepatitis B, scabies, tinea) [3]. Other diseases may be more serious, such as malaria, typhoid and tuberculosis; such problems should to be detected early after arrival [3]. During follow-up of adopted children, several types of pathology that are related to adoption or are more frequent in adopted children should be detected, including fetal alcohol spectrum disorder, early puberty, obesity, and behavioural problems (lack of concentration, agitation, etc.) [4]–[7]. However, health-care practitioners, especially general practitioners (GPs), need to be made aware that their role should not be confined to the detection of pathologies that originate from the adoptee's country of origin. They must also provide support to adoptive families from the beginning of the adoption project through the long period of development of the child.

Adoption carries with it a number of potential difficulties in addition to those seen in biological families [2], [4]–[7]. Parents who adopt are generally relatively old (in 2010, 92% of French adoptive parents were over 35 years old and 66% were over 40 years old) and tend to be childless couples [1], [8]. Such adoptive parents may be unprepared for looking after a mature child (and note that 41% of French international adoptees are more than 3 years old), who often arrives with a previous, sometimes tough life history, their own culture and habits, and may have health or psychological problems [2], [4]–[7].

Adoptees must face major changes in their life condition that require cognitive and emotional adjustment. Such adjustment is a key factor in the outcome of adoption that may condition the attachment of the adoptee to their new family [2]. However, it is a very complex and poorly understood process. Several factors may affect cognitive and emotional adjustment, as well as the attachment process, including previous abuse and neglect, malnutrition, exposure to drugs and alcohol, stigmatization and the feeling of loss that persists throughout the adoptee's life in different forms [2]. The age at adoption and the conditions of placement before adoption also play a crucial role; placement disruption is more frequent for older adoptees and in adoptees who are placed from foster care [2]. However, the history of the adoptive family is also an important factor to consider in the attachment process. A history of infertility, support from adoptive grandparents, long delay between placement and legal finalization of adoption, and a positive attitude towards the country of origin and the biological family may interact with the outcome [2]. Helping to build bridges between different life histories (those of the adoptee, the adoptive parents and the biological parents) may be one of the most important goals for health-care providers and may increase the chance of a successful adoption [9]. Failure of the attachment process may lead to dramatic events, such as legal re-abandonment, which occurs in about 1% of adoptions, and child abuse, which may occur when parents cannot face the behavioural or developmental problems of their child [8], [10]. It may also condition the future life and behaviour of the adoptees when they become adolescents [11].

Given that GPs meet families with adopted children relatively rarely, they may feel uneasy or lack resources when they have to manage the associated medical and psychological problems. The main aim of the study reported herein was to describe the problems faced by families before, during and after the arrival of their internationally adopted child. The secondary objective was to assess the usefulness of a specific medical structure, which could be a resource for the GP.

## Methods

### Research design

To develop a comprehensive understanding of such a complex experience as international adoption, and because quantitative methods are not well-suited to assessing families' perceptions of their needs and experiences, we used a qualitative approach that comprised conducting individual, semistructured guided conversations [12]. This method allows detailed exploration of individual experiences and may enable trust to be built that allows examination of a delicate topic that deals with intimacy and private life. The number of interviews was determined by the principle of data saturation [12].

All families in the Puy de Dome department (France) that had adopted internationally in 2003 were eligible for the study. Parents who adopted a child born in France or who refused to participate in the study were excluded. The year of adoption (2003) was chosen arbitrarily in order to interview families with substantial experience (6 years) but with limited memory bias. An information mailing was sent to the families in October 2008 and contact was made by telephone in February–October 2009. After consent, families were recontacted to arrange a meeting. Written informed consent was collected first, and a medical questionnaire was completed, in order to collect information about the pathologies diagnosed on arrival and during follow-up, as well as the investigations performed. Finally, an individual face-to-face interview was conducted and tape-recorded.

The study was approved by the Ethics Committee of the Rhône-Alpes. The interviews were conducted and recorded by one of two GPs at the parents' home. A discussion guide containing a predetermined set of topics was designed previously by the research team, on the basis of a literature review and preliminary interviews of three families and one medical doctor. After analysis of the three preliminary interviews, the discussion guide was modified by the research team.

At the beginning of the interview, the parents were asked to talk freely about their experience of adoption. Subsequently, the themes of the topic guide were introduced, including the adoption project, the arrival of the child and their integration, scholarship, behaviour and the family's needs after adoption. The children were not interviewed. At the end of the interview, the parents were asked to give their consent for the researchers to contact the GP or paediatrician responsible for care of the child. The individuals were contacted and asked about their feelings about adopted children with respect to assessment on arrival and medical follow-up.

### Data analysis

The tape-recorded interviews were transcribed verbatim for qualitative analysis, which was performed by the two investigators, assisted by two anthropologists. The other authors acted as co-readers. Data analysis was guided by the discussion guide and the general principle of an anthropological analysis, in which coding categories are developed inductively from the interviews [12]. After several readings of the corpus, themes were extracted independently from the transcribed interviews by each of the investigators, in an individual analysis. The results were compared between the investigators and new analyses were generated in an iterative process.

## Results

### Population studied

**Figure 1:**
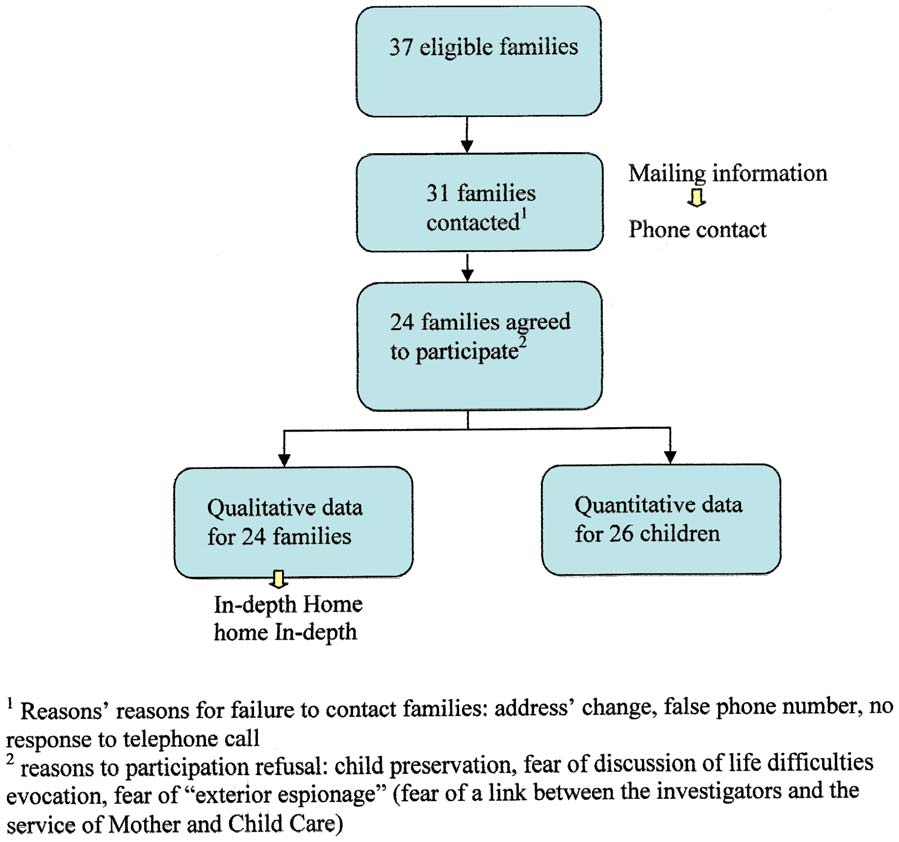
Set up of the study. Thirty-seven families who adopted in 2003 in the department of Puy de Dome, France were eligible for the study.

Thirty-seven families were eligible for the study (Figure 1). We failed to contact six families because of changes of address or telephone number. Seven families refused to participate. The reasons for refusal to participate included protection of the child, fear of discussing life difficulties, and fear of “exterior espionage” in the form of a link between the investigators and the service of Mother and Child Care. After exclusions, 24 families were interviewed, for a median time of 45 minutes (Figure 1). Data saturation was estimated to be achieved at 20 interviews, and was confirmed by four additional interviews. The characteristics of the families are described in Table 1.

**Table 1:** Characteristics of the 24 families who agreed to participate in the study.

*Characteristics*	*24 families*
**Married couples %**	89
**Single (women) %**	11
**Mean age (years) of parents at child arrival**	
Fathers	38.5 years
Mothers	38.5 years
**Parents' occupation**	
Housewife, %	4
Self-employed, %	20
Salaried, %	60
Teacher, %	16
**Age (years) of the child at arrival**	
***<1 year old***	
Real age^1^, %	39
Initially desired age^1^, %	42
***2 to 3 years old***	
Real age^1^, %	23
Initially desired age^1^, %	39
***>3 years old***	
Real age^1^, %	38
Initially desired age^1^, %	19
**Origin of the child**	
Asia, %	12
Eastern Europe, %	24
Latin America, %	19
Haïti, %	19
Africa, %	26

1 The real age is the age of the adopted child on arrival; the desired age refers to the age that the parents wanted when they started the adoption process.

### How the decision is made to choose adoption

The most frequent reason for choosing adoption was infertility; 14 couples had a long history of medical assisted conception (MAC). For these, the interviews aroused a great deal of emotion in the mothers, who admitted that this period was a physical and mostly a psychological ordeal, in which MAC was viewed only as a technical science with little place for humanity and emotional support: *“I'm not angry with the medical staff… They are technical, that's their job, but at the same time, they are not psychologists”; “We are only numbers”; “Somebody with less character than I, it would break them”*. Families also experienced psychological resistance to stopping the procedure of MAC after several years of unsuccessful attempts: *“It wasn't the choice we made and then it's difficult to reach the milestone and to imagine that one won't have a child who comes from yourself or your husband”.* For infertile couples, the decision to adopt is also made difficult by the fear that the parent–child relationship won't be established and *“the fear of not loving him”*. Most of the time, the decision to adopt was made after meeting friends, acquaintances or relatives who had experimented with adoption, rather than by following a suggestion made by the MAC medical staff. In fact, the adoptive couples stated that MAC staff did not make such suggestions: *“Specialists in MAC never talk about anything other than medical techniques”.*

In some cases, the reason for adoption was to continue with life as a single person, combined with the desire for a child. Other couples adopted when they already had a natural child, either because they were experiencing difficulty in having a second child, or because the mother did not want to experience pregnancy for a second time, or because she had had experienced problems with delivery the first time and did not want to repeat the experience.

### The long route to international adoption

Obtaining agreement for adoption was described by most families as an ordeal; they had the feeling that they must justify their desire to be parents. Moreover, the administrative procedures were experienced as being complicated. The information documents provided were experienced as being too dense and complex, whereas the oral information provided was insufficient and sometimes contradictory.

During the application process, prospective parents must determine how many children they can and want to adopt, and they may express certain restrictions on the gender, phenotype and health condition (medical and the carrying of disabilities) of the child they project to adopt.

Phenotype was a major criterion used by families. Seven families felt reluctant to adopt a black child because they feared that it would be subject to racism from other members of the family or inhabitants of their village or city. Some wanted the adoption to *“be as invisible as possible”*, in the sense that others would not perceive the child as being different in any way. At the opposite extreme, two families chose a black child at the initial phase of their project because they wanted *“to avoid any possible confusion with a biological one”*.

The health of potential adoptees was another major criterion. Most families refused children with special needs from the beginning of the procedure: *“We didn't want to endanger our family”; “We felt unable to educate a child who would have problems at the beginning”*. However, some of them faced a dilemma when they received a proposal for a handicapped child when they had refused a child with special needs in their initial project: *“Here we had a terrible Thursday because we had to say if we would take him or not […] my husband helped me because I couldn't say no […] It was a tough episode.”*

Criteria used by families that were of lesser importance were age and gender. All wanted a child *“as young as possible”* because older children were considered *“at risk”*. The age of the adopted child on arrival was compatible with the one desired by the family in 81% of cases (Table 1). The vast majority of families were indifferent to gender, except for two who wanted a girl, either to reduce the delays in the procedure (in China, girls are less difficult to adopt) or to have a girl after a previous boy.

The families had chosen international adoption because they perceived the procedures as quicker and because they may not meet the criteria to be allowed to adopt nationally. After obtaining agreement, the family has to choose the country from which the adoptee would come. The most important reason for choosing a particular country was the knowledge of a relative or friends who had successfully adopted from this country and who may provide help with local contacts and/or information about the administrative process. Other reasons that may have determined the choice included the existence of a relative who originated from the country (or another similar link), the phenotype of the child, and the knowledge that the time needed to complete the adoption procedure is shorter in certain countries.

The majority of families chose to apply on their own, rather than engage the services of Accredited Adoption Bodies (AABs are private-law legal entities that act as intermediaries for the adoption of minors under 15 years old). This was because being admitted to the AABs is associated with lengthy procedures, selective criteria such as age limitations and the proposal of children with special needs or older children, which may not fit with the initial ideas of the future parents. In support of their choice, families who used AABs said that they did not want to have any doubt about the legality of the adoption and that they needed support to go through the administrative process and to organize their stay in the adoptee's country.

Generally, the financial compensation for the adoption was fixed at the beginning, with no subsequent problems. However, several families, who adopted in Eastern Europe, reported corruption to an amount of at least 11 000 Euros to pay such persons as go-betweens, translators, and legal representatives. Several also had doubts about the real necessity for repeated expensive administrative procedures or repeated journeys. Some families said, without any prompting, that the cost would prevent them from starting a new process of adoption.

The adoption process after obtaining agreement lasted from 1 to 4 years, a period that was perceived as quite short for most of the families, especially for those who had experienced MAC: *“Once decided, it goes fast”.*

### First contacts and attachments

Some transitory reactions of fear or shyness were reported, but generally these lasted for only a few days: *“[…] he was very panicked, he was afraid because I was white […]. Therefore I didn't manage to hug him. He screamed as soon as I touched him […]. It lasted 3 days”.* When several journeys to the country of origin were needed, the separation was traumatic for both parents and child: *“When I came back to get him, he was sulking […]. It was horrible. It lasted at least four to five days. It was as if I had neglected him. He had this feeling of abandonment.”* After the first few days, sometimes after a few hours, a phase started in which the behaviour of parents and child became progressively more relaxed: *“He adopted us quickly and we adopted him quickly.”* However this progressive process of becoming attached was sometimes affected by episodes of doubt and guilt: *“[…] At the beginning, I felt a little bad, because he was attached only to his mother […]. Daddy didn't exist… And then, he went to daddy. Daddy, daddy…all the time. There was no mom then!! It was tough!”; “In the first period, it was tough because he was angry about being here […]. He was clearly unhappy; […] I realized that […] what we were offering him at that time did not suit him.”*

### Routine life

Many families reported difficulties for their child when going to bed, sleep with frequent waking, and nightmares for 1 month to 2 years (in one family with two children, the adoptee woke up every 90 minutes for 2 years). Physical contact was felt to be necessary for sleeping and many parents fell asleep with their child, arguing that they needed more closeness, given their history, or that they were not used to falling asleep alone in their previous life: “*[…] we are occidental, one decides that children sleep at the opposite side of the house […] whereas in most countries, they* [parents] *sleep with their children…”.*

Hyperphagia was reported in 10 children during the first few months. Restricting food was sometimes problematic and gave rise to a feeling of guilt. Some needed the help of a paediatrician to control the diet and the weight of their child. Three children had a follow-up examination by a doctor because of excess weight. *“He was always hungry. All the time, all the time…”; “We felt as though we were restricting his freedom, because we didn't allow him to eat outside meals…”*

Learning French was not a problem for any child and a level that allowed basic understanding and speaking was achieved in about 3 to 6 months. Apart from one child who remained at home until they were 6 years old, all the children who arrived in France before they were 3 years old went to school at the usual age of 3 years old. Older children tended to be sent to school soon after arrival because parents thought that their child was looking for contacts with other children who reminded them of their previous life in an orphanage: *“We realized that he was very keen on contact with other children […]. We thought that he had lived with plenty of kids… He must miss them. So we registered him at school.”*

Most families were proud about the school results of their child (*“brilliant”, “excellent”*), but some reported problems of concentration and attention with *“hyperactivity and disruption”.* Two of them required a life scholar assistant at school but, overall, none of the families reported their child having repeated a grade between 2003 and 2009.

Six black children out of 11 received discriminatory remarks related to the colour of their skin, especially at school: *“stool colour”, “you're not pretty because you're brown”, “mud colour”, “little black blooding sausage”*. Some children asked their parents if they could have white skin so that they would look like their parents. Reactions to discriminatory remarks were sometimes aggressive, with violence against other children, but, in contrast, some avoided other children or preferred to stay in the background. Two families reported that grandparents had been apprehensive about the foreign origin of the child: *“She* [grandmother] *was afraid that the children would be ill, badly educated, or be this or that…”.*

### Behaviour and, relationships

Most of the time, the parents reported no peculiar problems with the behaviour or comportment of their child. All but one came to feel very quickly that they were the child's parents, even while recognising clearly that they were not the biological parents: *“And I can't say my adopted child, because he's my son”*; *“It was always clear, we are not the ones who conceived him, we are his parents, we are those he loves, who are to help him to grow, to learn life.”* The relationship was characterized by a strong need by the child for demonstrations of affection; they frequently asked for cuddles or proof of love: *“There is a threat of abandonment: she has periods of anxiety, during which she takes the phone: Mum, come and pick me up.”* Some kept their distance without looking for physical contact: *“That's a child who never had loving cuddles and it was very difficult to give him a cuddle! And that's still the case; if he gives us a cuddle it's in secret!”*

Frequent behaviours reported were fits of anger, when there was a feeling of injustice or the child was upset, sometimes with a violent attitude (cries, hurtful words, breaking objects, aggressive behaviour). For the vast majority of children, these reactions were reported during the first few months following arrival and did not last. *“She became hysterical, I don't know how to describe it, at the slightest vexation, even without vexation.”* Some children remained highly irascible and two still have follow-up examinations with a child psychiatrist for behavioural problems (one for pyromania, one for violence).

The relationship between the two parents may be altered by the procedure of adoption or the arrival of the child. Three couples admitted to being over-focused on their child to the detriment of their own life as a couple, but they perceived such behaviour as *“normal because we are parents”*. Two experienced a conjugal crisis during the adoption process but said that they had not experienced any relationship problems since the arrival of the child. Two other couples divorced after having adopted, without a clear link with the adoption. Generally, the couples said that they were complementary: *“we are a team”.*

Many families estimated that, finally, *“adoption wasn't so terrible”* and that the media too frequently paint a black picture of this experience. However they admitted that they had to cope with many difficulties in the face of which they often felt alone. Two attitudes when confronted with difficulties were reported. Some did not look for help or did not think about it, sometimes because it reflected on their ability to be parents: *“[…] we had to go ahead, there were two kids to educate, to feed, […] we were parents, we had to be up to the mark.”* Others tried to seek help. Many consulted a child psychiatrist, most of the time in order to be reassured with respect to the normality of their child. Even if parents claimed to try *“to be as close as possible to biological parents”*, they maintained an increased level of vigilance with regard to behaviour or health problems, even minor ones, related to their adopted child.

### Pathologies diagnosed, medical investigations after adoption

The parents were asked to describe the first medical consultation with the adopted child. The consultation was performed by a paediatrician for 58% and a GP for 42% of the participants; 92% of the consultations were done during the first month after the arrival of the child. Of the children, 27% (n = 7) had no blood test performed, including one for whom the mother refused any kind of test. The recommended investigations that were not performed are described in Table 2. At the time of arrival, several types of pathology were detected: three parasite-related cases of diarrhoea (one with trichocephalosis, one with ascariasis; one with both amoebiasis and giardiasis; the children originated from Haiti and Ethiopia), five cases of scabies (from Haiti, Russia and Vietnam), four cases of tinea (one *Trichophyton soudanense*, three *Trichophyton tonsurans*) and two of impetigo. Two cases of scabies and two of tinea were diagnosed late after arrival and had been transmitted to the other family members. The GPs referred the children to a specialist (mainly dermatologists and psychiatrists) in 11 cases (scabies, tinea, intestinal parasites, early puberty, sleeping or behavioural problems). During follow-up examinations, three children, from Brazil, Ethiopia and Haiti, presented with obesity. One girl developed early puberty 4 months after arrival and one had symptoms consistent with fetal alcohol syndrome.

**Table 2:** Investigations recommended but not performed on arrival for 26 adopted children.

Test	% not performed
Serology Syphilis	
HIV	73%
Hepatitis A	42%
Hepatitis B	81%
Hepatitis C	42%
Vaccine	50%
	100%
Blood count	38%
Mantoux test	31%
Chest X-ray	69%
Stool culture, parasites	65%

### Families' expectations

Several families admitted to feeling helpless on arrival of the child and appreciated the support of their medical doctor: *“I needed to be reassured”; “We were lucky to be supported by our doctor”.* Eleven families took the initiative to consult a psychologist or a psychiatrist for reassurance: *“Some time after their arrival, we were a little confused… with this problem of sleeping: and so we asked for an appointment with a psychiatrist […] But sometime after, I realised that in fact young children are often afraid at night […] And finally, if I had had just a little help, just to tell me: it's normal, it has nothing to do with adoption, it would have been nice…”*; *“When you are the parent of an adopted child you tend to be over-focused, to be over-concerned, but perhaps too much […]”.* Most families viewed the idea of a specific consultation for adopted children favourably: *“Because the questions of health, sleeping, food… at first, it [the consultation] can be an interesting place where families find attention, a listening ear and advice.”* Some families also thought that meeting a specialist may be of interest before travelling and when the child arrived: *“It would be great to be able to talk with a doctor before travelling who could explain to us the risks of diseases there are in the country in which we'll adopt”.*

### Opinions of GPs and paediatricians

Eleven paediatricians and 18 GPs (all the doctors who cared for children included in the study) were interviewed about the usefulness of a specific consultation for adopted children. Eight paediatricians and 14 GPs were in favour of such a consultation and were interested in having contact with a specialist in case of problems related to adoption. Of these, six would refer only if there were problems, so as to avoid stigmatization. Three paediatricians and four GPs did not think that such a consultation would be useful: they did not consider it to be necessary in their medical practice or thought that these consultations might stigmatize the children.

## Discussion

We conducted individual interviews with 24 families who had adopted internationally in a French department in 2003. The interviews took place in the parents' home and allowed the interviewees free expression to describe the experience of adoption. Our study showed that the history of these families, from the beginning of the adoption project to its completion, is complex and deserves to be analysed carefully in order to understand better the relationship and the process by which the adoptive parents and adopted child form attachments. Health care providers should not only consider the history of the child, but should also try to piece together the puzzle of a tripartite history that involves the adoptive parents, the adoptee and the biological parents. They may help to build or consolidate bridges between the histories of the parents and the adoptee that may favour mutual attachment and prevent further trouble during the development of the child [9]. We also showed that adoptive parents have similar fears or transient difficulties that may be resolved quickly by listening and reassurance. Even if international adoption usually leads to a successful family bond, most families would support the development of a specific medical structure, which could be a resource for their GP [13]. However, the health-care providers interviewed were divided on the subject, and it is important to underline that such a structure should not replace the role of the GP but should be viewed as an additional support for families who may feel isolated during the adoption process [8]. The health-care providers also expressed a fear that a special consultation structure could be stigmatizing to children and families.

During MAC, parents should be informed about the possibility of adoption and its related difficulties. This information is part of a moral support system that was cruelly lacking for the parents who were interviewed in this study. Based on knowledge of what parents want and what they are able to do, another source of support would involve advising and informing families to guide them in developing their project of adoption. This step is critical for avoiding extremely serious subsequent problems, such as the re-abandonment of a child after adoption. During the adoption procedure, medical help should be offered that informs parents objectively about the pathologies cited in the medical record of the child. This requires the clinician to have experience and knowledge of relevant procedures in every country involved in adoption in order to be able to interpret the content of the medical record from the country of origin [8].

A medical evaluation has to be made soon after the arrival of the child. This evaluation should not only involve medical examination but should also include an assessment of the history of parents and the child and the conditions of the union between them. This cannot be achieved with a consultation that lasts less than one hour. The most difficult part of the consultation is to detect signs that could indicate that the parents and the adopted child will not have a natural and easy route to mutual recognition as parents and son or daughter [14]. For instance, the gap between pre-adoption expectations and the reality of the child has to be analysed carefully [8], [15]. This consultation has three other objectives. The first is to detect pathology, such as that caused by infectious diseases that could be severe (tuberculosis, malaria, etc.) or could spread [16] (hepatitis A for instance) or that may become chronic (hepatitis B, HIV, etc.). Parents must be prepared for the eventuality of unexpected incurable diseases that may be diagnosed at the time of arrival and that may affect the attachment process. The second aim is to determine the serological status of the child against viruses in order to administer appropriate vaccinations [17]. The third aim is to reassure the parents about their performance. Many parents may have doubts about their own abilities to become father and mother, and some may be confused when they have to face attitudes, behaviour or reactions they cannot categorize as normal, abnormal or related to adoption. Simple advice about food, sleep and general behaviour is often the best form of assistance that can be provided to these new parents. The GP, who knows the family best, plays a crucial role in this. In some cases, adopted children may need a specific follow-up examination for more thorough medical and/or psychological assessment. In this case, parents need to be supported to face the difficulties related to a disabled child, for which they may be unprepared.

Our study has a number of limitations. First, it was conducted in a single region in France and the families may be not representative of all the adoptive families in France or worldwide. Second, the study was not designed to assess problems related to adolescence. Further qualitative studies are required to address this very important topic [18]. Third, some information may have been missed because some families who refused to participate might have had more difficulties than those who participated. However, none of the 38 eligible families had severe problems, such as child abuse or re-abandonment. Fourth, some medical data in our study were quantitative and the small size of our study population may render them less valid. However, these medical aspects have been described already by several authors and confirmed in samples of larger size.

Since 2003, certain aspects of international adoption have changed and the children available for international adoption tend to be older and to be children with special needs. This will mean that adoption is associated with greater risk of failure and difficulties for which families have to be prepared and trained. This may be solved in part by the provision of practical information about this population and in part by development of a network that links GPs, paediatricians and a correspondent who has wide experience of adopted children.
